# Combination-Feeding Causes Differences in Aspects of Systemic and Mucosal Immune Cell Phenotypes and Functions Compared to Exclusive Sow-Rearing or Formula-Feeding in Piglets

**DOI:** 10.3390/nu13041097

**Published:** 2021-03-27

**Authors:** Emily C. Radlowski, Mei Wang, Marcia H. Monaco, Sarah S. Comstock, Sharon M. Donovan

**Affiliations:** 1 Department of Nutritional Sciences, Dominican University, River Forest, IL 60305, USA; eradlowski@dom.edu; 2 Department of Food Science and Human Nutrition, University of Illinois, Urbana, IL 61801, USA; meiwang@illinois.edu (M.W.); monaco@illinois.edu (M.H.M.); 3 Department of Food Science and Human Nutrition, Michigan State University, East Lansing, MI 48824, USA; comsto37@msu.edu

**Keywords:** formula, prebiotic, lipopolysaccharide, immune

## Abstract

Combination feeding (human milk and formula) is common and influences immune development compared to exclusive breastfeeding. Infant formulas contain prebiotics, which influence immune development. Herein, immune development of combination-fed (CF), sow-reared (SR) and formula-fed (FF) piglets, and the effect of prebiotics was tested. Piglets (*n* = 47) were randomized to: SR, FF, CF, FF+prebiotic (FP), and CF+prebiotic (CP). FP and CP received formula with galactooligosaccharides and inulin (4 g/L in a 4:1 ratio). CF and CP piglets were sow-reared for until d5 and then rotated between a sow and formula every 12 h. On day 21, piglets received an intraperitoneal injection of lipopolysaccharide 2 h prior to necropsy. Immune cells from blood, mesenteric lymph nodes (MLN), and spleen were phenotyped. Classical (nitric oxide synthase) and alternative (arginase activity) activation pathways were measured in isolated macrophages. Serum IL-6 and TNF-α were measured by ELISA. SR piglets had lower (*p* < 0.0001) CD4^+^ T-helper cells and higher (*p* < 0.0001) B-cells in PBMC than all other groups. CP piglets had higher (*p* < 0.0001) arginase activity compared to all other groups. FF piglets had higher (*p* < 0.05) IL-6 compared to both CF and SR, but were similar to FP and CP. Thus, CF, with or without prebiotics, differentially affected immunity compared to exclusively fed groups.

## 1. Introduction

Breastfeeding is the optimal form of nutrition for the human infant [1,2,3]. The breastfeeding initiation rate in the U.S. is currently at 83.2%, however, the prevalence of exclusive breastfeeding declines over the first few months postpartum, reaching ~25% at 6 months of age [4]. Some of these infants are fully weaned to formula, while ~57% of infants receive breast milk supplemented with formula by 6 months postpartum [4]. Using the data in the 2020 Breastfeeding Report Card [4], we estimated that 58% and 35% of infants are combination-fed at 6 and 12 months, respectively. Mothers choose to supplement formula for a multitude of reasons, including the perception of inability to produce an adequate amount of milk for their infant, short duration of maternity leave or discomfort while breastfeeding [5,6,7].

It is well documented that differences in short- and long-term health outcomes exist between the exclusively breastfed and exclusively formula-fed neonates. Exclusively breastfed neonates have lower rates of morbidity and mortality compared to their exclusively formula-fed counterparts [1,2,3]. Exclusive formula-feeding is associated with an increased risk of common childhood ailments such as diarrhea and otitis media, or an acute ear infection, which is 100% higher among exclusively formula-fed infants compared to exclusively breastfed infants during the first six months of life [3]. Formula feeding is related to higher risk of developing chronic diseases, including, asthma, type 2 diabetes, and childhood obesity [1,2,3,8].

The poorer outcomes associated with formula feeding may be partially attributed to the absence of protective components found in human milk [9,10], which include antibodies, human milk oligosaccharides (HMO), immune cells and cytokines [11,12]. These bioactive components influence the neonate’s microbial colonization [13], as well as immune system maturation [14,15]. Infant formulas lack these components, and neonates who are exclusively formula-fed show a different pattern of gut microbial colonization and immune development [13]. Although combination feeding (breastmilk and formula) is a common method of infant feeding in the U.S. [4,16], there is a paucity of epidemiological, clinical or basic research describing how this method of feeding affects neonatal intestinal or immune development.

HMOs constitute the third most predominant nutrient of human milk after lactose and fat [17]. These oligosaccharides interact with the resident microbiota and, in turn, the immune system [12]. HMO have only recently become available in sufficient quantities to supplement infant formula [18]. Therefore, prebiotics have been commonly used to mimic the biological activities of the naturally occurring oligosaccharides in human milk [10]. Non-digestible oligosaccharides have beneficial effects on the infant, including modulating immune development [10]. There are many different prebiotics available for use in infant formula, with galactooligosaccharides (GOS) and inulin being two of the most commonly used [10,19]. The prebiotics used in this study are both generally recognized as safe by the Food and Drug Administration and are already added to some infant formula. 

It is unknown whether mucosal innate immunity of infants receiving both breast milk and infant formula, with or without added prebiotics, more closely resemble their exclusively breastfed or formula-fed counterparts or whether these infants establish an intermediate balance between the two. This study aims to fill the gap in knowledge by examining the effects of combination feeding on neonatal immune development. We hypothesized that combination-fed piglets would mount a similar immune response to sow reared piglets, rather than formula fed, after being administered lipopolysaccharide. To undertake this study, our laboratory recently developed a novel piglet model that simulates combination feeding [20]. 

## 2. Materials and Methods

### 2.1. Experimental Design and Sample Collection

**Figure 1:**
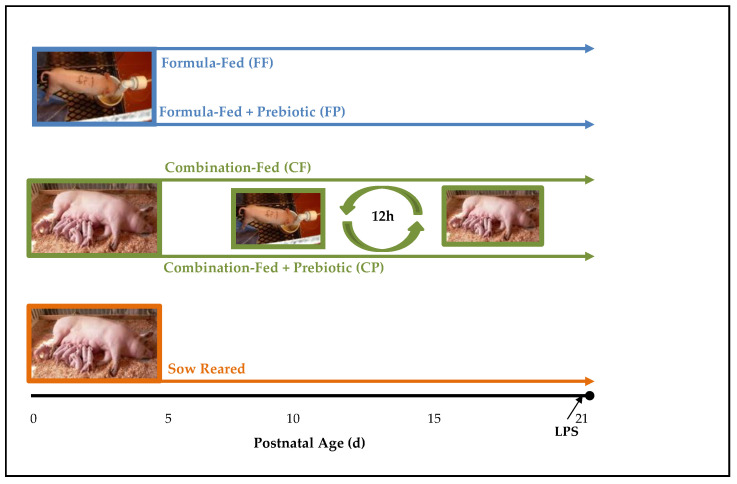
Study design. All piglets in all groups were vaginally delivered and remained with the sow for ~4 h. Piglets were then randomized into five experimental groups: sow-reared (SR, *n* = 9), formula-fed (FF, *n* = 12), formula-fed with prebiotics (FP, *n* = 10), combination-fed (CF, *n* = 8), or combination-fed with prebiotics (CP, *n* = 8). SR piglets remained with sows during the entire study. FF and FP piglets were removed from the sow after 4 h. CF and CP piglets remained with two sows (4–5 piglets per diet for each sow) for the first 5 day postpartum, then were rotated between nursing sows and formula feeding every 12 h. The formula was a non-medicated, commercial sow-milk replacer. Galactooligosaccharides (GOS, PURIMUNE^TM^) and agave inulin (BIOAGAVE^TM^) were added to the formula at a total concentration of 4 g/L in a 4:1 ratio. An i.p. LPS dose (10 µg/kg BW) was given 2 h before euthanasia on day 21.

Details of the experimental design and animal use are described in detail by Wang et al. [20] and are outlined in Figure 1. Briefly, piglets (*n* = 60) were vaginally delivered and remained with the sow for ~4 h to receive colostrum. Forty-seven of the piglets were randomized into 5 experimental groups: sow-reared (SR, *n* = 9), formula-fed (FF, *n* = 12), formula-fed with prebiotics (FP, *n* = 10), combination-fed (CF, *n* = 8), or combination-fed with prebiotics (CP, *n* = 8). SR piglets remained with sows during entire study. FF and FP piglets were removed from the sow after 4 h and were orally administered sow serum (5 mL/kg body weight [BW]) at 12 h and 24 h postpartum to provide additional passive immunity. The formula was a non-medicated, commercially available sow-milk replacer (Advance Liqui-Wean; Milk Specialties, Eden Prairie, MN (Table S1) that was reconstituted at 183 g powder/L. Two prebiotics, GOS (PURIMUNE^TM^) and agave inulin (BIOAGAVE^TM^), were purchased from GTC Nutrition (Golden, CO) and were added to the formula at a total concentration of 4 g/L in a 4:1 ratio. FF and FP piglets self-fed from a bowl and formula was provided at a rate of 360 mL/kg BW divided equally into 22 feedings/day by a timer-driven pump. The nutrient composition of the formula was previously described [21]. CF and CP piglets remained with sows (4–5 piglets per diet on each sow) for the first 5 day postpartum, then were rotated between nursing sows and formula feeding every 12 h. The litter size was maintained at 12–13 piglets per sow (day 1 to day 5) and 8–9 piglets per sow (day 6 to day 21). When the piglets were formula-fed, they were housed individually in metabolic cages at 25 °C with supplemental heat (30 to 32 °C) provided by radiant heaters suspended above the cages. To acclimate the piglets to be combination-fed, beginning on postnatal day 2, the piglets were placed in the cages for 1 h and the time was increased 1 h each day on day 3 and day 4. During the acclimation phase, rehydration solution (2.6 g/L NaCl, 1.5 g/L KCl, 13.5 g/L glucose anhydrous, 2.9 g/L sodium citrate dehydrate) was supplied in a bowl so that the piglets could learn to drink from the bowl. Piglet BW was recorded each morning and overall wellness assessed as previously described [22]. On day 21, all piglets were injected intraperitoneally (i.p.) with 10 µg/kg body weight of LPS (*Escherichia coli* serotype K-235; Sigma-Aldrich, St. Louis, MO, USA) 2 h prior to euthanasia. The work of Webel et al. [23] was utilized to design this aspect of the study, however, since the pigs in that study were older, a pilot study to determine appropriate LPS dosage and peak response time was conducted (data not shown). After 2 h, piglets were first sedated with Telazol (7 mg/kg body weight, Pfizer Animal Health, Fort Dodge, IA, USA) and then euthanized by intracardiac injection of sodium pentobarbital (Fatal Plus: 72 mg/kg body weight; Vortech Pharmaceuticals, Dearborn, MI). Blood samples were collected for serum separation by centrifugation (15 min, 3200× *g*, 4 °C) and immune cell isolation, as described below. Serum was frozen at −80 °C. Spleen and mesenteric lymph nodes (MLN) were excised for immune cell isolation, as described below. 

### 2.2. Isolation of Mononuclear Cells from Blood, Spleen and MLN

Peripheral blood mononuclear cells (PBMC) were isolated as described by Boudry and colleagues [24]. Briefly, 10 mL of heparinized blood was diluted in 25 mL of RPMI and the PBMC were recovered after centrifugation (400× *g*, 30 min) across a density gradient on lymphocyte separation medium (Ficoll-Paque PLUS, GE Healthcare, Uppsala, Sweden) [24]. The isolated PBMC were placed in culture medium (RPMI; Gibco Invitrogen, Grand Island, NY, USA) including 20% fetal calf serum (Gibco Invitrogen), 2 mM L-glutamine, 100 μg/mL penicillin, 100 mg/mL streptomycin, and 2 mM gentamycin). Mononuclear cells from MLN and spleen were obtained by dicing each organ sample into small pieces that were further dissociated using a gentleMACS™ Dissociator (Miltenyi Biotec, Bersgisch Gladbach, Germany) with three incubations at 37 °C of 30 min each in Hank’s Balanced Salt Solution (HBSS) plus Collagenase D (Roche Life Sciences, Indianapolis, IN, USA). The cells were filtered through a 100 and 40 μm filter (BD Biosciences, Franklin Lakes, NJ, USA) to form a single cell suspension. The number of viable cells was assessed by counting after staining with trypan blue (Gibco Invitrogen) and reported per mg of tissue [24]. 

### 2.3. Enrichment of Macrophages

After cells were counted, as referenced in Section 2.2, a portion of the cells were resuspended at 1 × 10^7^ cells per mL in complete RPMI media (Gibco Invitrogen). Ten mL of this solution were plated on 100 mm tissue culture dishes (BD Falcon 353003) and incubated for 16 h at 36 °C. After incubation, non-adherent cells were collected by pipetting off the liquid from the dishes and adding it to a 50 mL conical tube with the addition of 1 mL 20% fetal calf serum per 50 mL of solution. The tissue culture dish was then washed with room temperature PBS/EDTA. Adherent cells were collected by adding 2–3 mL of Trypsin-EDTA to each dish, followed by incubation at 37 °C for 10 min. After this incubation step, the liquid from the plates was collected and added to 50 mL conical tubes with 1 mL of 20% fetal calf serum. The plates were then washed with 10 mL of PBS/EDTA and the liquid was collected and placed into 50 mL conical tubes. The washing step was repeated for a total of two washes and all liquid collected. 

Both non-adherent and adherent cells in 50 mL conical tubes were centrifuged for 7 min at 740× *g* at 4 °C. The supernatants were discarded and the remaining cell pellets were washed twice with 10 mL PBS/EDTA and then resuspended in 5 mL PBS/EDTA. Cells were then counted after staining with trypan blue (Gibco Invitrogen).

### 2.4. Phenotypic Identification of Mononuclear Cells by Flow Cytometry

Mononuclear subpopulations were monitored by flow cytometry using a panel of fluorescein (FITC) or phycoerythrin (PE)-labeled monoclonal antibodies (mAbs) for dendritic cells (DC), macrophages (Mφ), natural killer (NK) cells, B-cells, and T-cells by established methods in our laboratory [25,26] and described below.

*T-Lymphocytes and Natural Killer Cells*: T-lymphocytes were identified by mouse anti-pig CD3 (PE-Cy5, Clone PPT3; Southern Biotech, Birmingham, AL, USA), mouse anti-pig CD4 (FITC, Clone 74-12-4) and mouse anti-pig CD8 (PE, Clone 76-2-11) antibodies (BD Biosciences, San Jose, CA, USA) [23]. Ten microliters of each antibody were added to 1 × 10^6^ cells from each sample. Samples were incubated for 20 min and then washed twice with PBS. The immune cell phenotypes and relative abundances were assessed by BD™ LSR II flow cytometry unit (BD Biosciences). NK cells were evaluated using percentage of cells identified as CD3^−^CD4^−^CD8^+^. The relative percentage of T-lymphocyte sub-populations, single-(CD4^+^, CD8^+^) or double (CD4^+^CD8^+^) positive cells, were also evaluated using FlowJo 7.0 software (FlowJo, Ashland, OR, USA). 

*B-Lymphocytes*: B-lymphocytes were identified using mouse anti-pig CD21 (PE, clone BB6-11C9.6, Southern Biotech, Birmingham, AL, USA) and mouse anti-pig MHCII (FITC, clone 2E9/13, ABD Serotec, Raleigh, NC, USA) antibodies [24]. Ten microliters of each antibody were added to 1 × 10^6^ cells from each sample. Samples were incubated for 20 min and then washed twice. The samples were then assessed by flow cytometry and the relative percentage of B-cells were evaluated using FlowJo 7.0 and expressed as a percentage of lymphocytes that were CD21^+^MHCII^+^. Lymphocytes, and the sub groups, were defined by FSC/SSC properties.

*Dendritic Cells and Macrophages*: DC were identified by mouse anti-pig CD172a (biotin, clone BL1H7, ABD Serotec; Strep-PECy5, BD Biosciences), mouse anti-pig CD16 (PE, clone G7, AbD Serotec), MHCII (FITC, clone 2E9/13, ABD Serotec) [24]. Mφ were identified by CD172a (Biotin, clone BL1H7, ABD Serotec), CD163 (RPE), CD14 (FITC, clone 74-12-4) and CD3 (PECy5) antibodies (BD Biosciences). Ten microliters of each antibody were added to 1 × 10^6^ cells from each sample. Samples were incubated for 20 min and then washed twice. The samples were then assessed by flow cytometry and the relative percentage of DC and Mφ were evaluated using FlowJo 7.0 software and expressed as a percentage of monocytes. Monocytes were defined by FSC/SSC properties, then identified as CD172a high. That population was then further separated through gating into dendritic cells (CD172a^+^, CD16^+^, MHCII^+^, CD3^−^, CD21^−^, CD163^−^) and macrophages (CD163^+^, CD172a^+^, CD14^+^, CD3^−^) and the percentage measured

### 2.5. Macrophage Activation Assays

*Nitric Oxide Production*: Isolated macrophages from spleen and MLN were plated into a 96-well plate at a concentration of 200,000 cells per well. A standard curve was prepared (0 mM–100 mM) by diluting sodium nitrite with double distilled water. Then, 100 µL of the Griess reagent (5% H_3_PO_4_, 2% sulfonamide, 0.2% naphthyl-ethyl-enediamine-dihydrochloride) was added to the cells and incubated at room temperature for 15 min [27]. Optical density at 540 nm was determined using a microplate reader (Molecular Devices, Spectra Max M2, Sunnyvale, CA, USA) and compared to the standard curve to estimate nitric oxide production. 

*Arginase Activity*: The assay was based on the protocol described by Gonçalves et al. [28]. Isolated macrophages from spleen and MLN were plated into a 96-well plate with a concentration of 200,000 cells per well. Then, 100 µL Triton 0.1% was added to lyse cells. After cell lysis, 100 µL of 50 mM Tris-HCl, pH 7.5 and 10 µL MnCl_2_ were added. Samples were transferred to safelock tubes and incubated at 56 °C to activate the enzyme. Arginine solution (100 µL, 0.5 M, pH 9.7) was added and samples were incubated for 2 h. Reactions were stopped using 800 µL of an acid mix of H_3_PO4, H_2_SO_4_, and H_2_O in a ratio of 1:3:7. α-isopentyl-S-thiolodiphosphate (α-ISPP) 6% (40 µL) was added, the tubes vortexed, incubated at first at 95 °C followed by a 4 °C incubation, for 30 min each. Samples were transferred to another 96-well plate and the optical density at 540 nm was determined using a microplate reader (Molecular Devices) and compared to a linear urea standard curve.

### 2.6. Serum IL-6 and TNF-α

Porcine-specific ELISA kits (R&D Systems, Minneapolis, MN, USA) for IL-6 and TNF-α were used to quantify serum cytokines and assays were conducted following manufacturer’s protocol. Serum samples were left undiluted. 

### 2.7. Statistical Analyses

Statistical analysis was first performed using one-way ANOVA according to the General Linear Model (GLM) procedure of SAS (SAS Institute, Cary, NC, USA). When significance was detected within the treatment, least significant difference test was used to identify differences between individual means. Statistical significance was set at *p* ≤ 0.05 and data are expressed as mean ± standard error of the mean (SEM).

## 3. Results

### 3.1. Phenotypic Identification of Mononuclear Cells by Flow Cytometry

**Figure 2:**
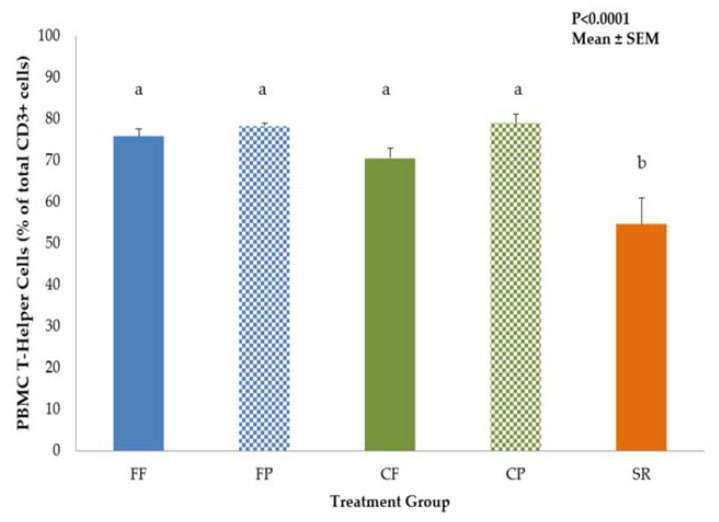
T-helper cells in PBMC of 21-day-old piglets fed different diets. SR had significantly lower percentages of circulating T-helper (CD3^+^/CD4^+^/CD8^−^) cells compared to all other diet groups. Values are mean ± SEM. Bars with different superscripts (e.g., a vs. b) indicate statistical significance at *p* < 0.05. Abbreviations: FF, formula-fed (*n* = 5); FP, formula+prebiotic (*n* = 7); CF, combination-fed (*n* = 8); CP combination-fed+prebiotic (*n* = 8); SR, sow-reared (*n* = 9).

**Figure 3:**
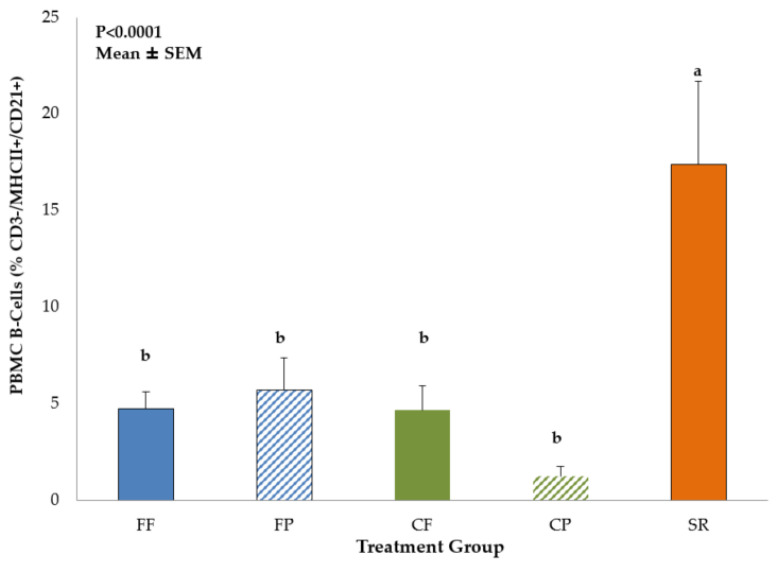
B-cells in PBMC of 21-day-old piglets fed different diets. SR had higher populations of circulating B-cells (CD3^−^/MHCII^+^/CD21^+^) than all other diet groups. Values are mean ± SEM. Bars with different superscripts (e.g., a vs. b) indicate statistical significance at *p* < 0.05. Abbreviations: FF, formula-fed (*n* = 12); FP, formula+prebiotic (*n* = 9); CF, combination-fed (*n* = 7); CP combination-fed+prebiotic (*n* = 8); SR, sow-reared (*n* = 7).

Data on immune cell populations in PBMC, MLN and spleen are shown in Supplementary Tables S2–S8. No significant differences in DC or Mφ populations among the dietary treatment groups were observed in PBMC, MLN or spleen. However, there was a dietary treatment effect on T-helper and B-cell populations in the PBMC. SR had significantly lower proportion of T-helper cell (CD3^+^CD4^+^CD8^−^) (Figure 2) and the higher proportion of B-cells cells compared to all other diet groups (Figure 3).

**Figure 4:**
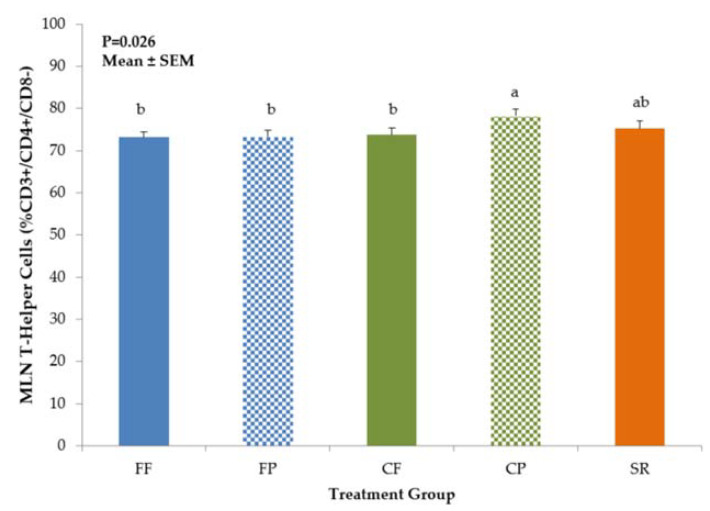
T-helper cells in MLN of 21-day-old piglets fed different diets. CP piglets had significantly higher populations of T-helper (CD3^+^/CD4^+^/CD8^−^) cells in their MLN compared to FF, FP, and CF groups, but was not different from SR. Values are mean ± SEM. Bars with different superscripts (e.g., a vs. b) indicate statistical significance at *p* < 0.05. Abbreviations: FF, formula-fed (*n* = 12); FP, formula+prebiotic (*n* = 10); CF, combination-fed (*n* = 8); CP combination-fed+prebiotic (*n* = 8); SR, sow-reared (*n* = 7).

**Figure 5:**
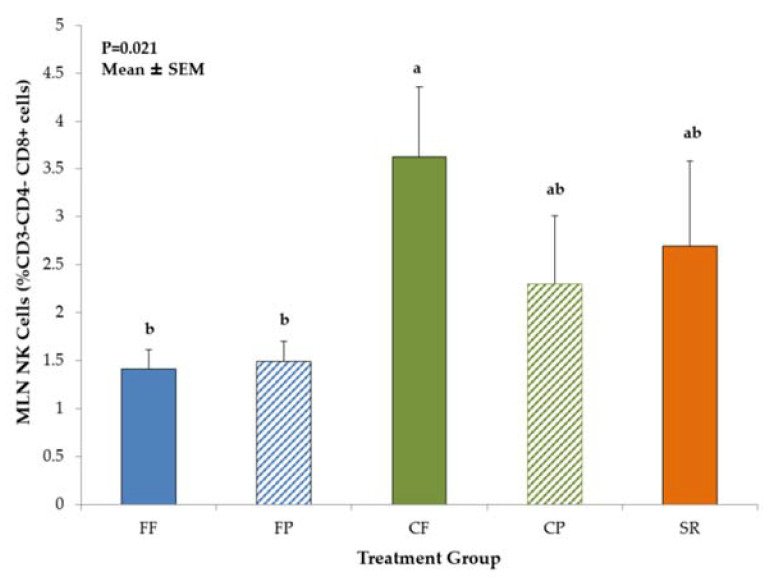
Natural Killer Cells in MLN of 21-day-old piglets fed different diets. The CF group had significantly higher population of NK cells compared to both FF and FP groups with both CP and SR being intermediate. Values are mean ± SEM. Bars with different superscripts (e.g., a vs. b) indicate statistical significance at *p* < 0.05. Abbreviations: FF, formula-fed (*n* = 10); FP, formula+prebiotic (*n* = 10); CF, combination-fed (*n* = 8); CP combination-fed+prebiotic (*n* = 7); SR, sow-reared (*n* = 6).

Diet-related differences in T-helper (CD3^+^CD4^+^CD8^−^) cell and NK cell (CD3^−^CD4^−^CD8^+^) populations were noted in the MLN, but not in the spleen. CP piglets had significantly higher T-helper cell populations compared to FF, FP, CF, but values were not different from SR (Figure 4). NK cell populations were significantly higher (*p* = 0.0214) in CF piglets than FF and FP piglets, with SR and CP being intermediate (Figure 5). 

### 3.2. Macrophage Activation Assays

**Figure 6:**
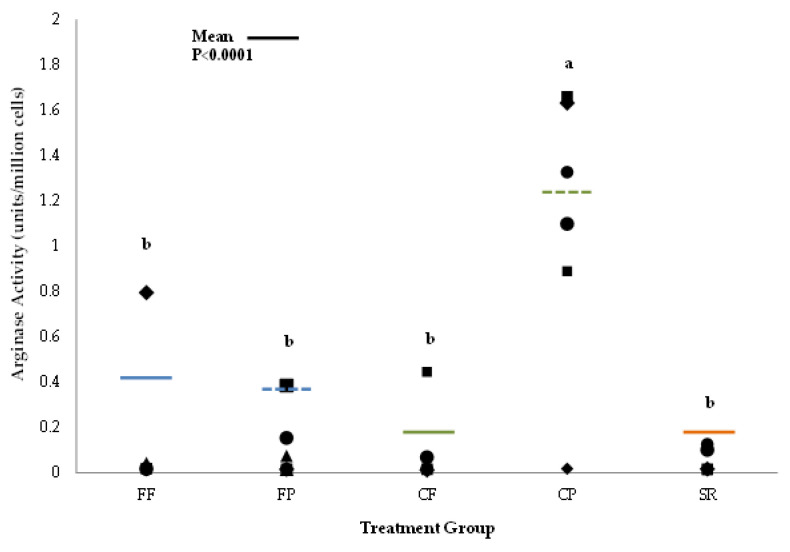
Arginase activity in macrophages isolated from spleens of 21-day-old piglets fed different Diets. Macrophages isolated from the spleen of piglets in the CP group had significantly higher arginase activity compared to all other groups. Values are mean ± SEM. Different superscripts (e.g., a vs. b) indicate statistical significance at *p* ≤ 0.0001. Abbreviations: FF, formula-fed (*n* = 8); FP, formula+prebiotic (*n* = 9); CF, combination-fed (*n* = 6); CP combination-fed+prebiotic (*n* = 6); SR, sow-reared (*n* = 6).

There were no differences in nitric oxide synthase (NOS) activity, a test for classical activation, in Mφ isolated from either spleen or MLN. There were also no differences among the groups in arginase activity, a marker for alternative activation, in Mφ isolated from MLN. However, Mφ isolated from CP spleen had higher (*p* < 0.0001) arginase activity compared to all other diet groups (Figure 6).

### 3.3. Serum IL-6 and TNF-α

IL-6 and TNF-α were measured in serum samples collected 2 h after LPS administration. Serum IL-6 concentration was highest in FF piglets and significantly different from both SR and CF (Table 1). The two prebiotic groups (FP and CP) were intermediate between FF, SR and CF. TNF-α did not differ among the diet groups (Table 1).

## 4. Discussion

The practice of supplementing breastfeeding with infant formula, or combination feeding, is reported to be the predominant method of feeding for the human infant by 6 months-of-age. [4]. Thus, investigating the effects of this feeding style on immunity development is warranted. In order to initiate this line of investigation, it was necessary to develop a new experimental model of combination feeding in the piglet [20]. Our goal was to mimic a feeding pattern wherein a mother may initiate exclusive breastfeeding and then move to combination feeding. Therefore, piglets were sow-reared for the first 5 d of life, before being randomized to combination feeding with or without prebiotic addition. 

In a companion paper, we reported that piglets in all dietary groups grew similarly over the duration of the study and had similar intestinal weight and lengths [20]. The data suggest that the combination feeding did not induce undue stress in the piglets that could have negatively impacted growth. However, differences in gut microbiome composition and volatile fatty acid concentrations were observed between SR, FF and CF piglets [20]. Bacterial composition, at the phylum level, in the colon of CF piglets was more similar to SR than FF piglets. Furthermore, there were no significant differences in bacterial genera between CF and SR piglets, while nine genera differed between CF and FF piglets including *Lactobacillus*, *Clostridium* XIVa, and *Fusobacterium*. Volatile fatty acid concentrations were similar between CF and SR, as well. While the addition of prebiotics has been shown to modulate microbial colonies in many piglet [29] and human infant studies [30,31], the combination of GOS and inulin did not have a strong impact, as the piglet microbiota clustered by overall feeding mode (SR, FF, CF). 

Given the importance of the gut microbiota in modulating early immune development [32], we hypothesized that immune development may differ between SR, FF, and CF piglets. Previous work investigating diet–host gene–microbiome interactions in breastfed and formula-fed infants, using the transcriptome of exfoliated epithelial cells and the fecal bacterial metagenome, demonstrated that the gut microbiota had an influence on specific genes related to microbial virulence and host immunity [33]. Genes related to bacterial-mediated reactive oxygen species signaling/epithelial homeostasis were up-regulated in breastfed infants and genes that prime mucosal inflammatory responses were up-regulated in formula-fed infants and down-regulated in breastfed infants [33]. Thus, mode of feeding had a direct influence on the “cross-talk” between the infant mucosal immune system and the microbiome. 

In this study, immune cell phenotypes (DC, Mφ, NK cells, T-helper, and B-cells) were examined to evaluate possible differences in immune development between the CF and exclusively SR-and FF-fed groups. Exposure to sow milk as well as prebiotics created a similar T-helper phenotype between CP and SR piglets in the MLN, but not in PBMCs. T-helper cells are part of the adaptive immune system and have subpopulations that are associated with differential responses to immune stimuli. Th1 T-helper cells elicit pro-inflammatory cytokines to further an immune reaction and recruit more immune cells and drive Mφ towards the classical activation pathway [34]. On the other hand, Th2 T-cells secrete more anti-inflammatory cytokines [35]. The balance between these subpopulations determines the quality of the immune response [36] and may be reflective of the maturity of the immune system in the infant [37,38]. The results we gained in both the PBMC T-helper profiles are similar to what was demonstrated in both Hawkes et al. [37] and Andersson et al. [38], with FF infants having higher CD4^+^ T-helper cell populations compared to breastfed. We also saw an increase in NK cells, a component of the innate immune system, in our SR and combined-fed groups, which is also similar to the findings for breastfed infants from Hawkes [37] and Andersson [38], except we saw this increase in MLN, not in PBMCs. However, the studies in human infants do not have access to the mucosal immune cells, therefore we cannot be certain that the NK cell populations are not higher in the MLN of breastfed compared to FF infants. In these two studies, the increase in NK cells and decrease in CD4^+^ T-cells was thought to be indicative of breastmilk’s effect in aiding the maturation of the breastfed infant’s immune system, likely attributed to the multitude of bioactive compounds that are naturally occurring. It was also hypothesized that the increase in CD4^+^ T-cells implied that FF infants are more likely to exhibit an adaptive immune response rather than an induction of tolerance when exposed to new antigens from diet or environment [38]. We also observed an increase in PBMC B-cells in the SR group. B-cells are also part of the adaptive immune system and work in concert with T-cells to produce an adaptive immune response [39]. B-cells are linked to the development and maintenance of the immune system, and are required for optimal T-cell activation during an immune response [39]. The increase in B-cell numbers observed in SR piglets may reflect a response to their environment (co-housed with the sow) that was amplified by the LPS stimulus. However, this was not observed in CF/CP piglets who spent 12 h per day with the sow. Perhaps more limited exposure to sow milk was not sufficient to induce greater B-cell proliferation in the combination fed groups. While we cannot draw the same conclusions as Hawkes [37] and Andersson [38] in regard to the immune development of mother-fed versus artificially reared neonates, the observed similarities confirm that early nutrition can impact development of both the adaptive and innate immune systems. It was interesting that CF/CP piglets exhibited an intermediate phenotype, with increased PBMC CD4^+^ T-cells and lower PBMC B-cells, similar to FF/FP, and increased NK cells in the MLN, similar to SR. Further investigation into these immune cell populations is needed to fully elucidate the more subtle effects of diet on these immune cell phenotypes. 

When looking more closely at Mφ activation, we hypothesized that SR, CF, and CP piglets would have more alternatively activated Mφ than FF and FP groups, due in part to their exposure to the immune cells and cytokines that are present in sow milk. Sow milk [40], like human milk [41], contains both TGF-β and IL-10, both of which promote immune tolerance. IL-10, in particular, encourages alternative activation of Mφ [42]. The arginase assay was used a measure for alternative activation of Mφ. Our arginase results show that in the spleen, CP piglets had significantly higher expression of alternatively activated Mφ compared to all other diet groups. The combination of exposure to sow milk and the prebiotic appeared to encourage more alternative Mφ activation. The SR and CF groups had relatively low levels of alternatively activated Mφ compared to the CP group. Sow milk alone was not sufficient to induce alternative activation, as hypothesized. 

As previously mentioned, an LPS challenge was used to elicit an immune response and elucidate any impact of diet on immune development. LPS binds to Toll-like receptor 4 (TLR4), which is present on the cell surface of Mφ and myeloid DC. TLR4 allows translocation and activation of the transcription factor NF-ĸB [43], which stimulates synthesis and secretion of cytokines (IL-6, TNFα) at the site of the infection, which will propagate the immune response [44]. Upregulation of inflammatory cytokine secretion such as IL-6, in response to pathogens has been linked to an increase risk for sepsis [45]. Our results show that diet may modulate the intensity of an innate immune response to LPS. FF piglets had the highest production of IL-6 compared to SR and CF piglets with FP and CP being intermediate. Lönnerdal et al. [46] measured plasma concentrations of various cytokines in breastfed and formula fed infants at 1, 4, and 6 months-of-age and reported significantly lower concentrations of IL-6 in breastfed versus formula-fed infants at each time point. In our study, the addition of prebiotics to the diet also tended to decrease the magnitude of the IL-6 response to LPS compared to exclusively FF piglets. 

Many of the results observed herein can be extrapolated human infants and some of the supporting evidence we use confirms this. To our knowledge, this is the first attempt to recapitulate combination feeding in an animal model. While some of the observed findings are consistent with observations in human infants, we acknowledge a limitation of the animal model is the environmental conditions of the sow-reared piglets versus breastfed human infants, which may contribute to some of the microbial immune responses observed between the SR and CF piglets and their human counterparts.

## 5. Conclusions

The findings shown herein indicate that early nutrition influences the development of the immune system, particularly acute immune responses. We found that the immune system of a CF piglet may not ‘choose sides’ and mimic either one of the exclusive feeding group, but rather represents a hybrid between the two. In some systematic reviews and meta-analyses, infants who receive any human milk (e.g., ever vs. never) are classified as breastfed [47,48,49]. However, our data suggest that those infants receiving both human milk and infant formula should be considered as a distinct group from exclusively breastfed or formula-fed infants, due to differences in immune measures and microbiome composition [20]. The prebiotics added to both formula and combination-feeding groups did not have as much impact as originally hypothesized on the immune parameters tested in this manuscript nor the gut microbiota measures in the companion paper [20]. The three main dietary approaches examined in this study (FF, CF, SR) had more influence on the immune parameters tested and may have dampened any effects that the prebiotics may have contributed. In addition, there are many health recommendations or expected outcomes based on research where only the two exclusive feeding styles (fully breastfed or fully formula-fed) are represented, and these recommendations may not apply to the combination-fed group (prevalence of allergic disease, future risk of overweight or obesity, etc.). Clinical and epidemiological studies in which human infants who are combination-fed are considered distinctly from exclusive FF or BF infants are warranted. 

## Figures and Tables

**Table 1:** Serum IL-6 and TNF-α concentrations (pg/mL) 2 h following LPS injection ^1,2^.

Cytokine	FF	FP	CF	CP	SR
IL-6	902 ± 163 ^a^	548 ± 114 ^ab^	277 ± 132 ^b^	542 ± 162 ^ab^	456 ± 103 ^b^
TNF-α	6.3 ± 0.9	5.5 ± 0.9	3.8 ± 0.3	6.6 ± 1.2	4.5 ± 0.5

^1^ Mean ± SEM (FF *n* = 8, FP *n* = 8, CF *n* = 8, CP *n* = 8, SR *n* = 7). ^2^ Different letter superscripts within a row (e.g., a vs. b) indicate statistical significance at *p* < 0.05. Abbreviations: FF, formula-fed; FP, formula+prebiotic; CF, combination-fed; CP combination-fed+prebiotic; SR, sow-reared; LPS, lipopolysaccharide.

## Supplementary Materials

The following are available online at https://www.mdpi.com/article/10.3390/nu13041097/s1, Table S1: Diet composition; Table S2: T-cell phenotypes in PBMC; Table S3: T-cell phenotypes in MLN; Table S4: T-cell phenotypes in spleen; Table S5: Natural Killer Cell populations in PBMC, MLN, and spleen; Table S6: B-cell populations in PBMC, MLN, and spleen; Table S7: Macrophage populations in PBMC, MLN, and spleen; Table S8: Dendritic Cell populations in PBMC, MLN, and spleen.
