# Supplementary material for: Combination-Feeding Causes Differences in Aspects of Systemic and Mucosal Immune Cell Phenotypes and Functions Compared to Exclusive Sow-Rearing or Formula-Feeding in Piglets
Source: Nutrients. 2021 Mar 27;13(4):1097. doi: 10.3390/nu13041097 (PMC8065485; doi:10.3390/nu13041097)
Supplement: Supplementary file 1 [file nutrients-13-01097-s001.pdf]

Supplementary Table S1. Composition of Sow Milk Replacer Formula. [1]

Component	Per kg Powder	Reconstituted (g/L)
Lactose (g)	479.0	87.0
Crude Protein (g)	250.0	45.8
Crude Fat (g)	130.0	23.8
Ash (g)	66.0	12.1
**Amino Acids**		
Arginine (g)	6.7	1.2
Histidine (g)	4.7	0.9
Isoleucine (g)	14.5	2.7
Leucine (g)	25.0	4.6
Lysine (g)	22.	4.1
Methionine (g)	5.0	0.9
Phenylalanine (g)	8.2	1.5
Threonine (g)	18.1	3.9
Tryptophan (g)	6.9	1.3
Valine (g)	14.6	2.7
**Vitamins**		
Vit B1 (mg)	8.5	0.2
Vit B2 (mg)	27.3	5.0
Vit B6 (mg)	2.0	0.4
Vit B12 (µg)	73.7	13.7
Vit A (KIU)	44.0	8.1
Vit D (KIU)	12.1	2.2
Vit E (IU)	33.2	6.1
Vit C (mg)	121.9	22.3
Choline (g)	1.8	0.32
Pantothenic Acid (mg)	61.6	11.3
Niacin (mg)	47.4	8.7
Folic Acid (mg)	3.35	0.61
Biotin (mg)	0.26	0.05
**Minerals and Electrolytes**		
Calcium (g)	6.2	1.1
Chloride (g)	8.1	1.48
Cobalt (mg)	1.23	0.23

[1] Advance LiquiWean, Milk Specialties, Eden Prarie, MN

Supplementary Table S2. T cell phenotypes in the peripheral blood mononuclear cell (PBMC) population of 21-d-old piglets who were sow reared (SR), formula-fed (FF) combination-fed (CF) or fed with prebiotics (FP and CP).

**Treatment Group**	**T Cell Population (% of CD3+ Events)**		
	T helper (CD3+CD4+CD8-)	Cytotoxic (CD3+CD4-CD8+)	Memory* (CD3+CD4+CD8+)
FF	75.8 ± 2.6[a]	3.7 ± 1.91[a]	7.3 ± 1.5
FP	78.3 ± 2.9[a]	1.6 ± 0.07[b]	5.6 ± 0.5
CF	70.5 ± 3.3[a]	2.5 ± 0.52[ab]	5.3 ± 0.4
CP	79 ± 3.3[a]	2.5 ± 1.41[ab]	5.8 ± 0.4
SR	54.6 ± 3.3[b]	1.9 ± 1.22[b]	7.1 ± 0.9

Values are expressed as means ± SEM

FF (n=5), FP (n=7), CF (n=8), CP (n=8), SR (n=9)

*indicates different 'n' for a group within a specific measurement. *FP (n=6), *CF (n=7)

Different letter superscripts in a column indicate statistical significance at $p \leq 0.05$

Supplementary Table S3. T cell phenotypes in the mesenteric lymph node (MLN) population of 21-d-old piglets who were sow reared (SR), formula-fed (FF) or combination-fed (CF) or with prebiotics (FP and CP).

Treatment Group	T Cell Population (% of CD3+ Events)		
	T helper (CD3+CD4+CD8-)	Cytotoxic (CD3+CD4-CD8+)	Memory (CD3+CD4+CD8+)
FF	73.1 ± 1.5[a]	6.6 ± 1.6	17.3 ± 2.4
FP	73.3 ± 1.5[a]	5.4 ± 1.5	18.3 ± 2.8
CF	73.7 ± 2.2[a]	4.8 ± 0.5	19 ± 2.9
CP	78.1 ± 2.4[b]	2.8 ± 0.7	16.9 ± 2.9
SR	75.2 ± 1.8[ab]	7.3 ± 1.3	12.3 ± 3

Values are expressed as means ± SEM

FF (n=12), FP (n=10), CF (n=8), CP (n=8), SR (n=7)

Different letter superscripts in a column indicate statistical significance at $p \leq 0.05$

Supplementary Table S4. T cell phenotypes in the spleen population of 21-d-old piglets who were sow reared (SR), formula-fed (FF) or combination-fed (CF) or with prebiotics (FP and CP).

**Treatment Group**	**T Cell Population (% of CD3+ Events)**		
	T helper (CD3+CD4+CD8-)	Cytotoxic (CD3+CD4-CD8+)	Memory (CD3+CD4+CD8+)
FF	70.6 ± 2.5	4.9 ± 0.9	11.3 ± 1.9
FP	71.2 ± 2.7	4.9 ± 1.2	12.7 ± 1.7
CF	66.1 ± 3.1	6.8 ± 1.5	13.2 ± 2.7
CP	74.1 ± 2.8	4.1 ± 0.9	12.9 ± 1.7
SR	75.1 ± 2.9	5.0 ± 1.0	7.9 ± 1.9

Values are expressed as means ± SEM

FF (n=12), FP (n=9), CF (n=7), CP (n=8), SR (n=7)

Supplementary Table S5. Natural killer cells (CD3-CD4-CD8+) in the PBMC, MLN and spleen population of 21-d-old piglets who were sow reared (SR), formula-fed (FF) or combination-fed (CF) or with prebiotics (FP and CP).

Treatment Group	Natural Killer Cells (% of CD3- Events)		
	PBMC	MLN*	Spleen
FF	5.42 ± 1.75	1.41 ± 0.70[b]	17.74 ± 2.78
FP	8.02 ± 1.71	1.49 ± 0.67[b]	16.55 ± 2.57
CF	9.84 ± 1.85	3.63 ± 2.06[a]	17.91 ±3.84
CP	9.39 ± 4.28	2.29 ± 2.00[ab]	14.68 ± 2.97
SR	14.07 ± 4.97	2.69 ± 2.17[ab]	11.44 ± 3.12

Values are expressed as means ± SEM

FF (n=12), FP (n=9), CF (n=7), CP (n=8), SR (n=7)

*indicates different 'n' for group within a specific tissue: *SR (n=6), *FP (n=10), *CF (n=8), *CP (n=7)

Different letter superscripts in a column indicate statistical significance at $p \leq 0.05$

Abbreviations: MLN, mesenteric lymph nodes; PBMC, peripheral blood mononuclear cells

Supplementary Table S6. B cells (CD21+MHCII+) in the PBMC, MLN and spleen population of 21-d-old piglets who were sow reared (SR), formula-fed (FF) or combination-fed (CF) or with prebiotics (FP and CP). The total lymphocytes gates were determined from forward scatter and side scatter plots after flow cytometric analysis.

Treatment Group	B Cells (% of Lymphocytes)		
	PBMC[#]	MLN*	Spleen**
FF	4.74 ± 0.86[b]	25.37 ± 3.45	10.56 ± 1.69
FP	5.69 ± 1.68[b]	24.26 ± 3.47	12.11 ± 2.17
CF	4.66 ± 1.23[b]	17.35 ± 3.53	7.12 ± 2.18
CP	1.23 ± 0.51[b]	22.16 ± 3.22	9.83 ± 1.66
SR	17.38 ± 4.32[a]	20.50 ± 7.18	7.25 ± 1.22

Values are expressed as means ± SEM

FF (n=12), FP (n=9), CF (n=7), CP (n=8), SR (n=7)

[#], *, ** indicates different 'n' for group within a specific tissue: [#]CF (n=6), [#]CP (n=7),*CF (n=8), *CP (n=7),**FP (n=10)

Different letter superscripts in a column indicate statistical significance at $p \leq 0.05$

Abbreviations: MLN, mesenteric lymph nodes; PBMC, peripheral blood mononuclear cells

Supplementary Table S7. Macrophages (CD163+, CD172a+, CD14+, CD3-) in the PBMC, MLN and spleen population of 21-d-old piglets who were sow reared (SR), formula-fed (FF) or combination-fed (CF) or with prebiotics (FP and CP). Differentiation of the macrophage cell type within total monocytes was determined from forward scatter and side scatter plots after flow cytometry analysis.

Treatment Group	Macrophages (% of Monocytes)		
	PBMC#	*MLN	Spleen**
FF	0.13 ± 0.05	0.17 ± 0.06	2.06 ± 0.23
FP	0.14 ± 0.11	0.17 ± 0.08	1.72 ± 0.18
CF	0.15 ± 0.09	0.16 ± 0.05	1.5 ± 0.36
CP	0.13 ± 0.05	0.17 ± 0.06	1.6 ± 0.81
SR	0.14 ± 0.02	0.16 ± 0.14	1.5 ± 0.31

Values are expressed as means ± SEM.

FF (n=4), FP (n=5), CF (n=6), CP (n=7), SR (n=4)

*, **,# indicates different 'n' for group within a specific tissue:*FF (n=8), *FP (n=6), **FP (n=3), **SR (n=3) **CF (n=5), #SR (n=2)

Different letter superscripts in a column indicate statistical significance at p≤0.05

Abbreviations: MLN, mesenteric lymph nodes; PBMC, peripheral blood mononuclear cells

Supplementary Table S8. Dendritic cells (CD172a+, CD16+, MHCII+, CD3-, CD21-, CD163-) in the PBMC, MLN and spleen population of 21-d-old piglets who were sow reared (SR), formula-fed (FF) or combination-fed (CF) or with prebiotics (FP and CP). Differentiation of the dendritic cell type within total Monocytes was determined from forward scatter and side scatter plots after flow cytometry analysis

Treatment Group	Dendritic Cells (% of Monocytes)		
	PBMC#	MLN	Spleen**
FF	12.7 ± 2.75	16.54 ± 1.96	33.13 ± 7.18
FP	13.3 ± 2.22	17.03 ± 1.88	27.6 ± 3.28
CF	11.3 ± 2.73	18.18 ± 2.81	28.41 ± 2.54
CP	13.3 ± 1.39	20.36 ± 1.82	26.6 ± 2.79
SR	10.2 ± 1.81	20.20 ± 2.64	36.3 ± 5.52

Values are expressed as means ± SEM.

FF (n=12), FP (n=10), CF (n=8), CP (n=7), SR (n=6)

*,# indicates different 'n' for group within a specific measurement: #FP (n=9), #CF (n=7), #CP (n=6), #SR (n=7), **FF (n=9), **CP (n=8, **SR (n=5)

Different letter superscripts in a column indicate statistical significance at p≤0.05

Abbreviations: MLN, mesenteric lymph nodes; PBMC, peripheral blood mononuclear cells
